# Can ChatGPT assist urologists in managing overactive bladders?

**DOI:** 10.1097/JS9.0000000000000887

**Published:** 2023-12-12

**Authors:** Mei-Lin Feng, Xiaoshuai Gao

**Affiliations:** a School of Food and Biological Engineering, Chengdu University, Chengdu; b Department of Urology and Institute of Urology (Laboratory of Reconstructive Urology), West China Hospital, Sichuan University, Sichuan, People’s Republic of China

*Dear Editor*,

Overactive bladder (OAB) is a syndrome that encompasses a range of symptoms, primarily marked by a persistent sense of urgency to urinate. This condition is frequently accompanied by increased frequency of urination, especially during the night (nocturia), and may also involve occasional urge incontinence. The impact of OAB on individuals’ daily lives and social engagements is substantial, making it a prevalent and burdensome ailment that affects a significant portion of the population. The management of OAB involves a variety of interventions, including behavioral therapy, medication, and neuroregulation techniques^1^. Chat Generative Pretrained Transformer (ChatGPT) is an advanced artificial intelligence model created by Open artificial intelligence (AI), leveraging cutting-edge deep learning technology. Designed to comprehend and generate human-like language, ChatGPT showcases a diverse range of capabilities, including answering straightforward queries, crafting resumes, offering dating guidance, and much more. In our exploration of ChatGPT’s clinical potential, we have observed that it can provide satisfactory responses to basic and uncomplicated medical inquiries. Leveraging its robust retrieval capabilities, ChatGPT demonstrates competence in furnishing accurate and acceptable information in the medical domain^2^. This study endeavors to investigate the supplementary role of ChatGPT in the pharmacological management of bladder hyperactivity, as well as its potential application in various areas such as lifestyle modification guidance, dissemination of information, support for treatment decision-making, management of adverse reactions, patient consultation, and overall support.

ChatGPT can provide guidance and support for lifestyle changes in patients with OAB. As an AI language model, ChatGPT can engage in interactive conversations, provide information, answer questions, and offer suggestions related to lifestyle modifications that can help manage OAB symptoms (Fig. 1).

**Figure 1:**
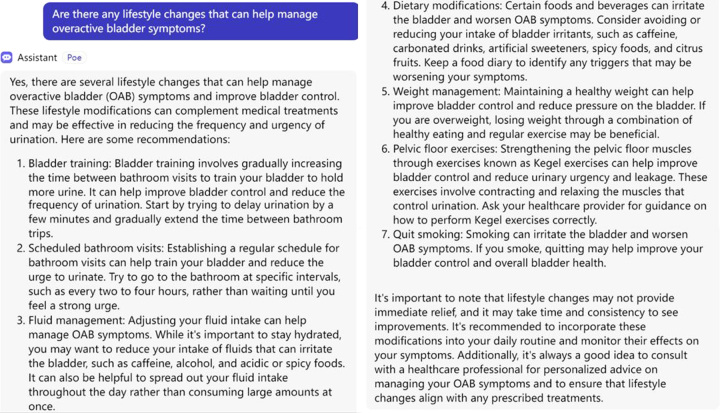
ChatGPT recommendations for lifestyle changes in overactive bladder patients.

In light of the expanding array of online resources and the escalating need for personalized medical information, ChatGPT presents a promising avenue for both patients and healthcare professionals engaged in the management of OAB. ChatGPT harnesses its natural language processing capabilities to engage in interactive conversations, furnish pertinent information, address inquiries, and offer guidance throughout the treatment journey^3^. Functioning as an educational tool, ChatGPT provides accurate and up-to-date insights regarding OAB, available medications, and their mechanisms of action. By leveraging ChatGPT’s capabilities, tailored support and enhanced decision-making can be facilitated, thereby optimizing the overall management of OAB.

Treatment decision support is an essential component, and ChatGPT can assist healthcare professionals in making informed choices regarding the most suitable pharmaceutical treatment, taking into account patient-specific variables^4^. Through comprehensive analysis of individual patient data, encompassing medical history and potential interactions with other medications, ChatGPT can offer valuable insights and recommendations to optimize treatment selection and facilitate dosage adjustments, thereby augmenting the quality of care provided. Achieving competency in judging the accuracy of diagnoses and treatment plans necessitates the accumulation of clinical experience and the thorough examination of a substantial body of literature. Without these essential components, it becomes exceedingly challenging to assess the correctness of diagnoses and treatment plans, thereby compromising the legitimacy and effectiveness of the entire diagnostic and treatment process.

Managing potential side effects poses a significant hurdle in the pharmacological treatment of OAB. In this regard, ChatGPT can serve as a valuable resource by offering information regarding potential adverse reactions, proposing strategies to mitigate their impact, and providing guidance to patients on when it may be necessary to seek medical advice or consider adjusting their medication dosage^4^. By equipping patients with this knowledge, ChatGPT empowers them to make informed decisions and actively participate in their treatment journey, ultimately enhancing their overall therapeutic experience.

Furthermore, OAB can significantly affect a patient’s emotional well-being. To address this aspect, ChatGPT can offer emotional support to individuals, respond to their inquiries, and provide coping strategies to help them effectively manage the psychological impact of OAB^5^. Moreover, ChatGPT can facilitate connections to additional resources or healthcare professionals who can offer further support if required. By providing this comprehensive support, ChatGPT strives to enhance the holistic care provided to patients, ensuring not only their physical well-being but also their emotional resilience throughout their journey with OAB.

In conclusion, this study aims to explore the auxiliary role of ChatGPT in the drug treatment of OAB. By investigating its potential applications in lifestyle modification instruction, information dissemination, treatment decision support, side effect management, and patient counseling and support, we can gain insights into how ChatGPT can augment the care provided to individuals with OAB. Understanding the capabilities and limitations of this technology will contribute to the development of innovative and patient-centered approaches in OAB management, ultimately improving treatment outcomes and enhancing the patient experience.

## Ethical approval

Not applicable.

## Sources of funding

Postdoctoral Fund of West China Hospital of Sichuan University (grant no. 2023HXBH041); Natural Science Foundation of Sichuan Province (grant no. 2023NSFSC1532).

## Author contribution

X.G.: writing original draft; M.-L.F.: review, editing, and supervision.

## Conflicts of interest disclosure

The authors declare no conflicts of interest.

## Research registration unique identifying number (UIN)

Not applicable.

## Guarantor

Mei-Lin Feng.

## Data availability statement

Data are available from the corresponding author if the requirement justified.
